# Single nucleotide polymorphisms unravel hierarchical divergence and signatures of selection among Alaskan sockeye salmon (*Oncorhynchus nerka*) populations

**DOI:** 10.1186/1471-2148-11-48

**Published:** 2011-02-18

**Authors:** Daniel Gomez-Uchida, James E Seeb, Matt J Smith, Christopher Habicht, Thomas P Quinn, Lisa W Seeb

**Affiliations:** 1 School of Aquatic and Fishery Sciences, 1122 Boat St NE Box 355020 Seattle, WA 98195-5020, USA; 2 U.S. Fish & Wildlife Service, Abernathy Fish Technology Center, 1440 Abernathy Creek Road, Longview, WA 98632, USA; 3 Division of Commercial Fisheries, Alaska Department of Fish and Game, 333 Raspberry Road, Anchorage, AK 99518, USA

## Abstract

**Background:**

Disentangling the roles of geography and ecology driving population divergence and distinguishing adaptive from neutral evolution at the molecular level have been common goals among evolutionary and conservation biologists. Using single nucleotide polymorphism (SNP) multilocus genotypes for 31 sockeye salmon (*Oncorhynchus nerka*) populations from the Kvichak River, Alaska, we assessed the relative roles of geography (discrete boundaries or continuous distance) and ecology (spawning habitat and timing) driving genetic divergence in this species at varying spatial scales within the drainage. We also evaluated two outlier detection methods to characterize candidate SNPs responding to environmental selection, emphasizing which mechanism(s) may maintain the genetic variation of outlier loci.

**Results:**

For the entire drainage, Mantel tests suggested a greater role of geographic distance on population divergence than differences in spawn timing when each variable was correlated with pairwise genetic distances. Clustering and hierarchical analyses of molecular variance indicated that the largest genetic differentiation occurred between populations from distinct lakes or subdrainages. Within one population-rich lake, however, Mantel tests suggested a greater role of spawn timing than geographic distance on population divergence when each variable was correlated with pairwise genetic distances. Variable spawn timing among populations was linked to specific spawning habitats as revealed by principal coordinate analyses. We additionally identified two outlier SNPs located in the major histocompatibility complex (MHC) class II that appeared robust to violations of demographic assumptions from an initial pool of eight candidates for selection.

**Conclusions:**

First, our results suggest that geography and ecology have influenced genetic divergence between Alaskan sockeye salmon populations in a hierarchical manner depending on the spatial scale. Second, we found consistent evidence for diversifying selection in two loci located in the MHC class II by means of outlier detection methods; yet, alternative scenarios for the evolution of these loci were also evaluated. Both conclusions argue that historical contingency and contemporary adaptation have likely driven differentiation between Kvichak River sockeye salmon populations, as revealed by a suite of SNPs. Our findings highlight the need for conservation of complex population structure, because it provides resilience in the face of environmental change, both natural and anthropogenic.

## Background

Disentangling the roles of geography and ecology driving population divergence and speciation has been a common goal among evolutionary biologists [1]. Several studies spanning diverse taxa suggest that the influence of these factors is often hierarchical: geography, landscape features, and vicariance may be important at larger spatial scales, whereas ecology and life history may be important at finer spatial scales (fishes: [2,3]; birds: [4,5]; mammals: [6]; plants: [7]). Hierarchical structure and divergence below the species level have key implications for conservation and the definition of evolutionary significant units [8]. For management applications, a hierarchical distribution of population-level diversity has been deemed critical for the resilience of commercially exploited species, which some authors have defined as biocomplexity [9-11]; hierarchical structure may also provide a strong buffer against interannual fluctuations in abundance or 'portfolio effect', therefore ensuring long-term sustainability of wild populations in an era of growing anthropogenic impacts [12].

Research on salmonid systems has greatly enhanced our understanding of hierarchical divergence; in fact, genetic variance is normally larger between salmon populations inhabiting different basins than between salmon populations from the same basin that differ in ecological attributes [2,3,13,14]. This configuration is likely a result of historical contingency (e.g., postglacial recolonization) and contemporary evolution of life history types [2,15]. Anadromous salmon populations are highly philopatric; adults return to their natal sites in freshwater to reproduce, thus promoting genetic isolation and local adaptation [16]. In particular, adult sockeye salmon (*Oncorhynchus nerka*, Walbaum 1792) spawn among tributaries, outlet rivers, beaches, and even glacial habitats [13,14,17] after typically spending 2 or 3 years in the ocean [18,19]. A combination of natural and sexual selection appears to maintain phenotypic divergence between populations, but especially between those using different types of spawning habitats (e.g., beaches and tributaries: [20,21]). In addition, the timing of spawning often differs systematically between habitat types, and the regularity in timing of migration and spawning is critical to the structure and conservation of populations [22-24].

Molecular tools have become instrumental for quantifying population divergence and reproductive isolation in applied evolutionary biology studies. Single nucleotide polymorphisms (SNPs) are now the marker of choice among many geneticists for addressing evolutionary questions [25-28]. The majority of studies using these abundant bi-allelic markers have hitherto focused on model organisms [26] with fewer applications to nonmodel taxa (but see [29-31]). Because SNPs can be linked to functional genes, it is important to determine which markers have been likely targets of selection, otherwise, estimates of gene flow may be compromised [32]. So-called 'genome scans' have enabled identification of putative markers under selection exhibiting larger or smaller estimates of divergence--often referred to as 'outliers'--than selectively neutral markers [33]. Outliers have been related to adaptive divergence in several studies [34-36]; nevertheless, demography and neutral processes can leave similar signatures to selection in the genome. Population bottlenecks or expansions can be mistaken for selective sweeps [33,37]. Furthermore, recent models of hierarchical structure suggest that, when gene flow occurs predominantly within rather than between groups of demes, the number of outliers could be upwardly biased [38].

**Figure 1:**
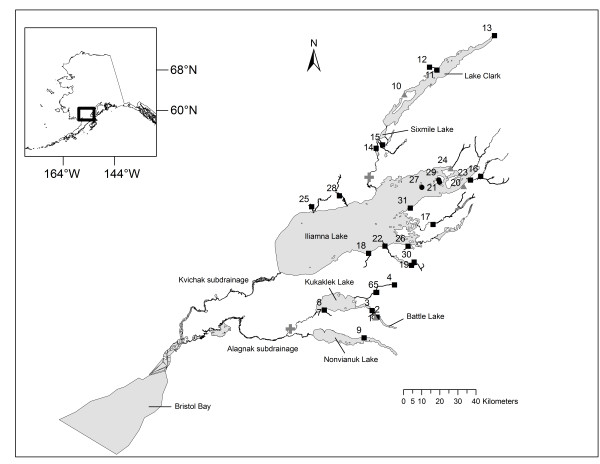
**Populations of sockeye salmon from the Kvichak River drainage**. Location in southwest Alaska appears in rectangle of upper left insert. Numbers correspond to populations within Alagnak (1 - 9) and Kvichak (10 - 31) subdrainages. Population legends--tributaries: squares; mainland beaches: triangles; and island beaches: circles. Grey crosses indicate two waterfalls that may represent velocity barriers for migrating adults.

Here we employed multilocus genotypes from 42 nuclear and three mitochondrial SNPs isolated for sockeye salmon [39-41] to typify 31 spawning populations throughout the Kvichak River, which drains into Bristol Bay, southwest Alaska (Figure 1; Table 1). Our primary goal was to assess the differential roles of geography (e.g., discrete boundaries or continuous distance) and ecology (e.g., spawning habitat and timing) driving the spatial distribution of genetic diversity as revealed by SNPs. The Kvichak River is divided into two major subdrainages, each containing multiple lakes (Figure 1; Table 1). Subdrainages and lakes should represent natural landscape boundaries driving genetic diversity within and between *O. nerka *populations because (i) they were likely recolonized at varying times through different founding events as ice sheets sequentially retreated at the end of the Late Wisconsin glacial maximum (*ca*. 25,000 - 10,000 years BP: [42]); (ii) lakes provide nursery habitat for juvenile sockeye salmon growth, and also, an opportunity for strong olfactory imprinting, a crucial aid for adult homing behaviour [19]; and (iii) they may be isolated by the presence of waterfalls (Figure 1) that delay or deter upstream migration of returning adults, especially during years with increased river runoff [43,44]. Lakes in turn harbour locally adapted populations that spawn among diverse environments, including mainland-beach, island-beach, and tributary habitats [45]. Some of these populations have discrete patterns of migration and spawn timing [23,24], which adds a temporal dimension to spatial divergence [46]. Both ecological attributes--spawning habitat and timing of reproduction--predict that dispersal is most likely to occur between populations spawning in the same habitat, at the same time, or both, and this has found empirical support [21,47-49]. Dispersal in salmonids may also follow an isolation-by-distance pattern if migration and drift have reached equilibrium [50], implying that gene flow is more efficient between nearby populations than those far apart [16]. We hypothesize that both geography and ecology should interact to influence large- (lake, subdrainages) and fine-scale dispersal (intralake) but we expected their importance to vary depending on the spatial scale: geographic attributes should be more important at larger scales, whereas ecological characteristics should be more important at finer scales.

**Table 1:** Ecological and genetic summary statistics* for sockeye salmon populations

Subdrainage/Population number	Location	Spawning habitat^†^	Spawn timing^‡^	Lake	*n*	*A*_R_	*H*_E_	HWE
Alagnak								
1	Battle Lake Beach	MBEA	4 September	Battle	192	1.822	0.227	0.445
2	Battle Lake Tributary	TRIB	4 September	Battle	192	1.809	0.228	0.711
3	Battle River	TRIB	4 September	Kukaklek	192	1.785	0.223	0.801
4	Funnel Creek (E)^§^	TRIB	16 August	Kukaklek	171	1.841	0.235	0.743
5	Moraine Creek	TRIB	5 September	Kukaklek	191	1.851	0.230	0.843
6	Moraine Creek (E)^§^	TRIB	21 August	Kukaklek	192	1.830	0.242	0.187
7	Nanuktuk Creek	TRIB	9 September	Kukaklek	192	1.861	0.238	0.293
8	Nanuktuk Creek (E)^§^	TRIB	15 August	Kukaklek	192	1.858	0.248	0.723
9	Kulik River	TRIB	4 September	Nonvianuk	192	1.887	0.253	0.696
Kvichak								
10	Chulitna Lodge Beach	MBEA	5 October	Clark	96	1.810	0.227	0.268
11	Kijik River	TRIB	20 September	Clark	96	1.794	0.226	0.768
12	Lower Kijik River	TRIB	30 September	Clark	96	1.850	0.237	0.656
13	Upper Tlikakila	TRIB	23 September	Clark	96	1.788	0.207	0.606
14	Newhalen River	TRIB	15 September	Sixmile	92	1.892	0.259	0.681
15	Tazimina River	TRIB	23 August	Sixmile	95	1.870	0.253	0.284
16	Chinkelyes Creek	TRIB	5 September	Iliamna	96	1.879	0.251	0.857
17	Copper River	TRIB	23 August	Iliamna	96	1.931	0.242	0.997
18	Dennis Creek	TRIB	26 August	Iliamna	96	1.911	0.256	0.756
19	Dream Creek	TRIB	5 September	Iliamna	95	1.932	0.252	0.998
20	Finger Beach	MBEA	20 September	Iliamna	84	1.869	0.250	0.998
21	Flat Island	IBEA	16 August	Iliamna	94	1.937	0.253	0.999
22	Gibralter River	TRIB	30 August	Iliamna	86	1.892	0.252	0.067
23	Iliamna River (L)^§^	TRIB	17 October	Iliamna	95	1.937	0.253	0.287
24	Knutson Beach	MBEA	6 October	Iliamna	94	1.932	0.246	0.857
25	Lower Talarik Creek	TRIB	28 August	Iliamna	165	1.898	0.254	0.057
26	Nick Creek	TRIB	27 August	Iliamna	96	1.979	0.253	0.331
27	Triangle Island	IBEA	7 August	Iliamna	96	1.895	0.248	0.970
28	Upper Talarik	TRIB	17 August	Iliamna	189	1.923	0.264	0.561
29	Woody Island	IBEA	16 August	Iliamna	96	1.918	0.256	0.729
30	Southeast Creek	TRIB	2 September	Iliamna	94	1.928	0.254	0.942
31	Tommy River	TRIB	30 August	Iliamna	96	1.835	0.248	0.920

*After excluding two outlier SNPs and three mitochondrial SNPs (see text for details). *n*, sample size; *A*_R_, allelic richness or number of alleles corrected for a minimum sample size of 76 diploid individuals; *H*_E_, unbiased expected heterozygosity; HWE, goodness-of-fit to Hardy-Weinberg Equilibrium expectations. ^§^E = early migrants; L = late migrants. ^†^MBEA = mainland beach; IBEA = island beach; TRIB = tributary. ^‡^Historical dates of peak spawning activity.

Our secondary goal was to evaluate the scope of neutral vs. adaptive differentiation in sockeye salmon as revealed by locus-specific estimates of divergence. Here we used two outlier detection methods to identify and characterize potential candidate SNPs responding to environmental selection, emphasizing which mechanism(s) may maintain the genetic variation of outlier loci. These analyses were conducted and reported first (see below) in order to avoid estimates of (neutral) divergence that may be biased by selection. Emphasis was placed on violations of the demographic assumptions of outlier detection models, including potential bottlenecks and hierarchical structure between populations [38,51]. We subsequently conducted exploratory analyses of Hardy-Weinberg and linkage disequilibrium, estimated genetic diversity and differentiation, and tested for the relative effects of geography and ecology on population divergence, using SNP sets that included or excluded these outlier loci.

## Results

### Detection and characterization of outliers

**Figure 2:**
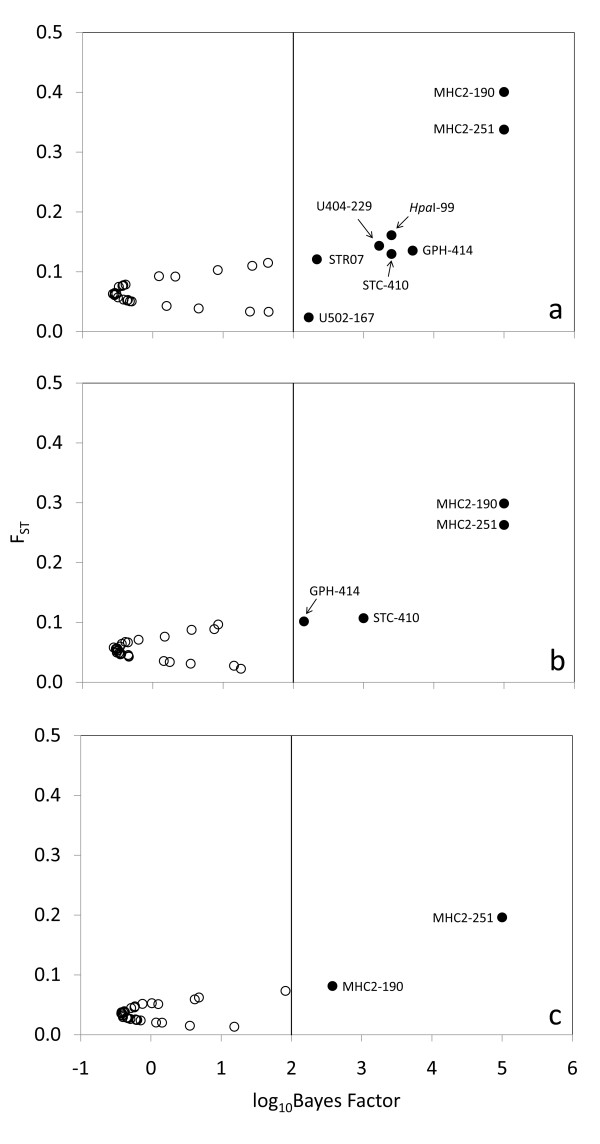
**Detection of outlier SNPs using BAYESCAN**. Populations included in the analysis: (a) the entire Kvichak River drainage, (b) after excluding Lake Clark, and (c) Iliamna Lake only. *F*_ST_: locus-specific genetic divergence among populations; log_10_Bayes Factor: decision factor in logarithmic scale (base 10) to determine selection; a vertical line indicates "decisive" evidence for selection. Filled circles represent candidates for selection; empty circles represent putatively neutral loci. Marker labels have been simplified (the prefix "*One_*" is missing).

**Figure 3:**
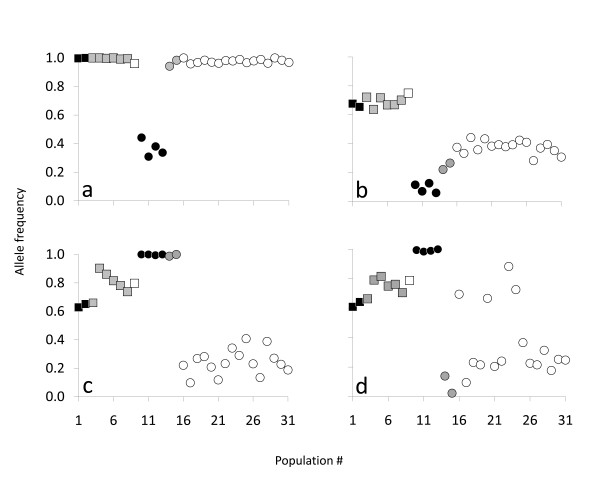
**Frequency plots of the major allele for four outlier SNPs in sockeye salmon**. SNPs correspond to (a) *One*_*HpaI-99*, (b) *One*_*GPH-414*, (c) *One*_*MHC2-190*, and (d) *One*_*MHC2-251 *genotyped across 31 Alaskan populations (see Table 1 for population codes). Squares represent lakes from the Alagnak subdrainage (Battle: black; Kukaklek: grey; Nonvianuk: empty), whereas circles represent lakes from the Kvichak subdrainage (Clark: black; Sixmile: grey; Iliamna: empty).

**Figure 4:**
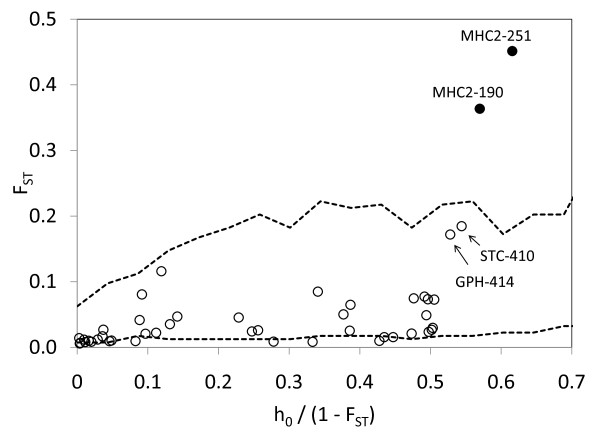
**Detection of outlier SNPs using Arlequin 3.5**. Assuming a hierarchical model of migration and excluding Lake Clark populations. *F*_ST_: locus-specific genetic divergence among populations; h_0_/(1 - *F*_ST_): a modified measure of heterozygosity per locus. Dashed lines indicate lower and upper 95% confidence intervals for variation in neutral *F*_ST _as a function of h_0_/(1 - *F*_ST_). Filled circles represent candidates for selection; empty circles represent putatively neutral loci. Marker labels have been simplified (the prefix "*One_*" is missing).

BAYESCAN [51] suggested seven candidates for diversifying selection and one candidate for balancing selection (Table 2; Figure 2a). It quickly became apparent, though, that the majority of outlier SNPs were driven by a few divergent populations from Lake Clark (Figure 1; Table 1). When Lake Clark populations were removed, four loci no longer appeared as outliers (Figure 2b); their allele frequencies were very similar and showed strong differences (>0.5) only between Lake Clark and the rest of populations (e.g., *One*_*HpaI-99*: Figure 3a). Lake Clark populations further showed the lowest estimates of diversity within populations (see *Results: Genetic diversity and differentiation*), possibly indicating presence of bottlenecks. Marked allele frequency differences between subdrainages (~0.5) characterized other outliers, such as *One_GPH-414 *(Figure 3b) and *One*_*STC-410 *(not shown), suggesting that gene flow was more predominant within subdrainages than between subdrainages. Simulations in Arlequin 3.5 [52] confirmed that *One_GPH-414 *and *One*_*STC-410 *were no longer outliers assuming hierarchical structure between subdrainages (Figure 4). Therefore, only *One _MHC2-190 *and *One_MHC2-251 *consistently appeared to be under diversifying selection (Figure 2a-c; Figure 4).

**Table 2:** Summary of outlier SNP loci* or candidates for diversifying^† ^or balancing^‡ ^selection in sockeye salmon

SNP locus	Description	SNP location	Reference
*One_GPH-414*^†^	Glycoprotein hormone alpha subunit	Intronic	Elfstrom *et al. *[40]
*One_HpaI-99*^†^	*Hpa*I repetitive elements	Intronic	Elfstrom *et al. *[40]
*One_MHC2-190*^†^	Major Histocompatibility Complex class II	Exonic	Elfstrom *et al. *[40] Miller & Withler [54]
*One_MHC2-251*^†^	Major Histocompatibility Complex class II	Intronic	Elfstrom *et al. *[40] Miller & Withler [54]
*One_STC-410*^†^	Ovarian stanniocalcin	Intronic	Elfstrom *et al. *[40]
*One_STR07*^†^	Unknown	-	Elfstrom *et al. *[40]
*One_U404-229*^†^	Unknown	-	Habicht *et al. *[41]
*One_U502-167*^‡^	Unknown	-	Habicht *et al. *[41]

*Using BAYESCAN [51] and Arlequin 3.5 [52].

Outlier SNPs were annotated to protein-encoding sequences with three exceptions (Table 2). Even though most mutations were intronic, *One_MHC2-190 *was located in exon β1 of the major histocompatibility complex (MHC) class II which corresponded to a nonsynonymous substitution (aspartic acid to tyrosine) in the putative antigen recognition site [53]. Only 61 nucleotides separated this and the paired locus *One_MHC2-251 *located within an intervening intron [54]. Interestingly, allele frequencies appeared uncorrelated between *One_MHC2-190 *and *One_MHC2-251 *for some populations (Figure 3c-d); for instance, they fluctuated greatly between Sixmile Lake populations (1.0 to 0.01). Also, a group of populations from the east end of Iliamna Lake (16-Chinkelyes Creek, 20-Finger Beach, 23-Iliamna River, and 24-Knutson Beach) had much higher frequencies than the remaining populations for *One_MHC2-251 *(Figure 3d), but not *One_MHC2-190 *(Figure 3c).

### Hardy-Weinberg equilibrium and linkage disequilibrium tests

No significant (*p *= 0.05) departures from Hardy-Weinberg equilibrium were observed in any population or locus following exclusion of three mitochondrial SNPs (Table 1). However, we detected highly significant linkage disequilibrium for 3 nuclear locus-pairs across populations after a sequential Bonferroni correction for *k *tests (*k *= 703, *p *< 7.1 × 10^-5^): *One_MHC2-190 *(*I *= 0.433) and *One_MHC2-251 *(*I *= 0.465); *One_Tf_ex10-7 *(*I *= 0.632) and *One_Tf_ex3-18 *(*I *= 0.084); *One_GPDH *(*I *= 0.668) and *One_GPDH2 *(*I *= 0.082). Based on the information content per locus, *I *(Shannon-Weaver's index: in parentheses above), both *One_MHC2-190 *and *One_MHC2-251 *were kept for subsequent analyses, while *One_Tf_ex3-18 *and *One_GPDH2 *were excluded from further analyses. Exclusion of these loci decreased the pool of nuclear SNPs from 42 to 40.

### Genetic diversity and differentiation

Estimates of SNP diversity and differentiation were obtained for three distinct nuclear sets of markers to account for possible effects of selection: 'No Outliers' or the putatively neutral set (38 SNPs: excluding *One*_*MHC2-190 *and *One*_*MHC2-251*), 'Outlier 1' (39 SNPs: including *One*_*MHC2-190*), and 'Outlier 2' (39 SNPs including *One*_*MHC2-251*). Differences between sets were emphasized if present; otherwise 'No Outliers' should be considered the default set.

Allelic richness (*A*_R_) ranged between 1.785 (3-Battle Lake River) and 1.979 (26-Nick Creek) and expected heterozygosity (*H*_E_) varied between 0.207 (13-Upper Tlikakila) and 0.264 (28-Upper Talarik Creek: Table 1). Both metrics were highly correlated across all populations (Spearman *R *= 0.733 *p *< 0.001). We also detected differences in *A*_R _(χ^2 ^= 21.2, *p *< 0.001) and *H*_E _between lakes (χ^2 ^= 21.5, *p *< 0.001). The lowest diversities were found within Clark and Battle, whereas the highest were in Sixmile and Iliamna (Table 3). Diversity contrasts between subdrainages (Alagnak vs. Kvichak) were also significant (*A*_R_: *U *= 22.5, *p *= 0.004; *H*_E_: *U *= 11.5, *p *= 0.012).

**Table 3:** Average estimates of genetic diversity* (± SE, standard error) from sockeye salmon hierarchical groups

Lake	Subdrainage	Mean *A*_R _± SE	Mean *H*_E _± SE	Number of Populations
Battle	Alagnak	1.815 ± 0.009	0.227 ± 0.003	2
Kukaklek	Alagnak	1.838 ± 0.028	0.239 ± 0.006	6
Nonvianuk	Alagnak	1.887 ± 0.000	0.253 ± 0.000	1
Clark	Kvichak	1.811 ± 0.021	0.213 ± 0.011	4
Sixmile	Kvichak	1.881 ± 0.004	0.257 ± 0.004	2
Iliamna	Kvichak	1.912 ± 0.040	0.245 ± 0.004	16

*Using 38 SNPs. *A*_R_, allelic richness; *H*_E_, expected heterozygosity.

Estimates of global divergence were slightly higher for 'Outlier 1' (*F*_ST _= 0.082; 95% CI: 0.047 - 0.123; *p *< 0.001) and 'Outlier 2' (*F*_ST _= 0.080; 95% CI: 0.047 - 0.118; *p *< 0.001) than for the 'No Outliers' set (*F*_ST _= 0.060; 95% CI: 0.041 - 0.095; *p *< 0.001). Pairwise *F*_ST _for the entire drainage were significantly correlated between SNP sets based on simple Mantel tests and Pearson correlation coefficient, *r *('No Outliers' vs. 'Outlier 1' *r *= 0.98, *p *< 0.001; 'No Outliers' vs. 'Outlier 2' *r *= 0.98, *p *< 0.001; 'Outlier 1' vs. 'Outlier 2' *r *= 0.97, *p *< 0.001). Global differentiation among mitochondrial SNPs was also highly significant (Φ_PT _= 0.054; *p *< 0.001) but lower than estimates found for nuclear SNPs.

Pairwise *F*_ST _values were consistently significant between populations located in different lakes for all three SNP sets, even though multiple population comparisons within Iliamna Lake suggested no differentiation (see additional file 1). Notable examples included: (i) 1-Battle Lake Beach and 2-Battle Lake Tributary, two populations that spawn in contrasting habitats but are in close geographic proximity; (ii) 5-Moraine Creek and 6-Moraine Creek Early, and (iii) 7-Nanuktuk Creek and 8-Nanuktuk Creek Early, which may represent either two discrete populations migrating at different times or one large population with a protracted spawning period.

### Effects of geography at large spatial scales (entire drainage)

Simple and partial Mantel tests suggested that geographic distance played a greater role than spawn timing influencing genetic distances for the entire drainage, albeit the effects of both variables were significant at this scale (Table 4). We also found strong evidence for hierarchical structure in both nuclear and mitochondrial SNPs through an analysis of molecular variance (AMOVA: see additional file 2); significance of variance components was found in all three SNP sets. Lakes harboured the highest percentage of genetic variance (6- to 13-times higher than between populations within lakes, depending on marker type), whereas subdrainage variance was similar to that found between populations within subdrainages. However, subdrainage structure became important after markedly differentiated Lake Clark populations were excluded. Yet, between-lake genetic variance was consistently higher than subdrainage variance, even when Lake Clark was excluded (additional file 2).

**Table 4:** Pearson correlations between genetic distances and two explanatory variables for sockeye salmon populations

	Simple†		Partial†	
	
	Geographic distance	Spawn timing	Geographic distance	Spawn timing
Entire drainage				
Neutral	0.427***	0.256*	0.445***	0.290*
Outlier 1	0.429***	0.251*	0.447***	0.286*
Outlier 2	0.403***	0.307*	0.428***	0.342**
Iliamna Lake				
Neutral	0.224*	0.020	-	-
Outlier 1	0.260*	0.040	-	-
Outlier 2	0.389**	0.763***	0.436***	0.775***

*p < 0.05, **p < 0.01, ***p < 0.001. †Mantel tests (see text for details).

**Figure 5:**
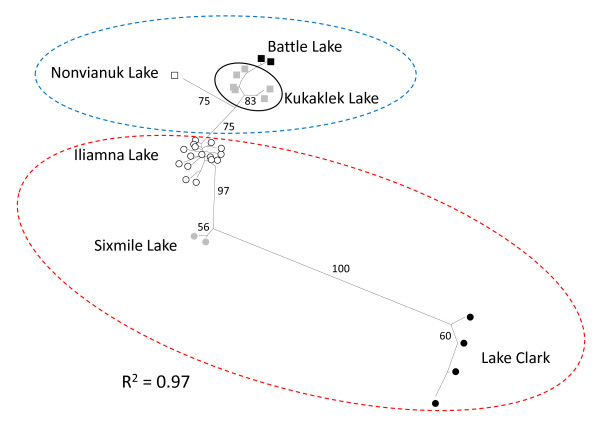
**Unrooted neighbour-joining tree between sockeye salmon populations**. Based on Cavalli-Sforza & Edwards [51] chord distances for 38 nuclear ('No Outliers') SNPs. Segmented lines indicate the Alagnak (blue) and Kvichak (red) subdrainages; squares represent lakes from Alagnak (Battle: black; Kukaklek: grey; Nonvianuk: empty squares), whereas circles represent lakes from Kvichak (Clark: black; Sixmile: grey; Iliamna: empty). Numbers on branches represent percentages (only >50% are shown) of bootstrap support after generating 1000 resampled trees. *R*^2 ^depicts how well branch length and the variation of the distance matrix are correlated.

An unrooted neighbour-joining tree using Cavalli-Sforza & Edwards [55] chord distances revealed that spatial structure was largely driven by marked differences between populations inhabiting distinct lakes, followed by less pronounced differences between populations within lakes (Figure 5); this result was consistent with the hierarchical AMOVA. Within the Alagnak subdrainage, a long branch separated Nonvianuk Lake from the rest of the populations; for the Kvichak subdrainage, Lake Clark showed the longest branch indicative of strong reproductive isolation. Also, a tree *R*^2 ^= 0.97 indicates that branch lengths explained a substantial amount of the variation present in the matrix of population distances [56].

### Effects of ecology at fine spatial scales (Iliamna Lake)

Simple and partial Mantel tests performed within the population-rich Iliamna Lake suggested that spawn timing had a higher degree of correlation with pairwise genetic distances than geographic distances, albeit only for the 'Outlier 2' SNP set (Table 4). The effects of geography were still evident at this scale, however, despite the fact that they exhibited lower correlation values (Table 4).

**Figure 6:**
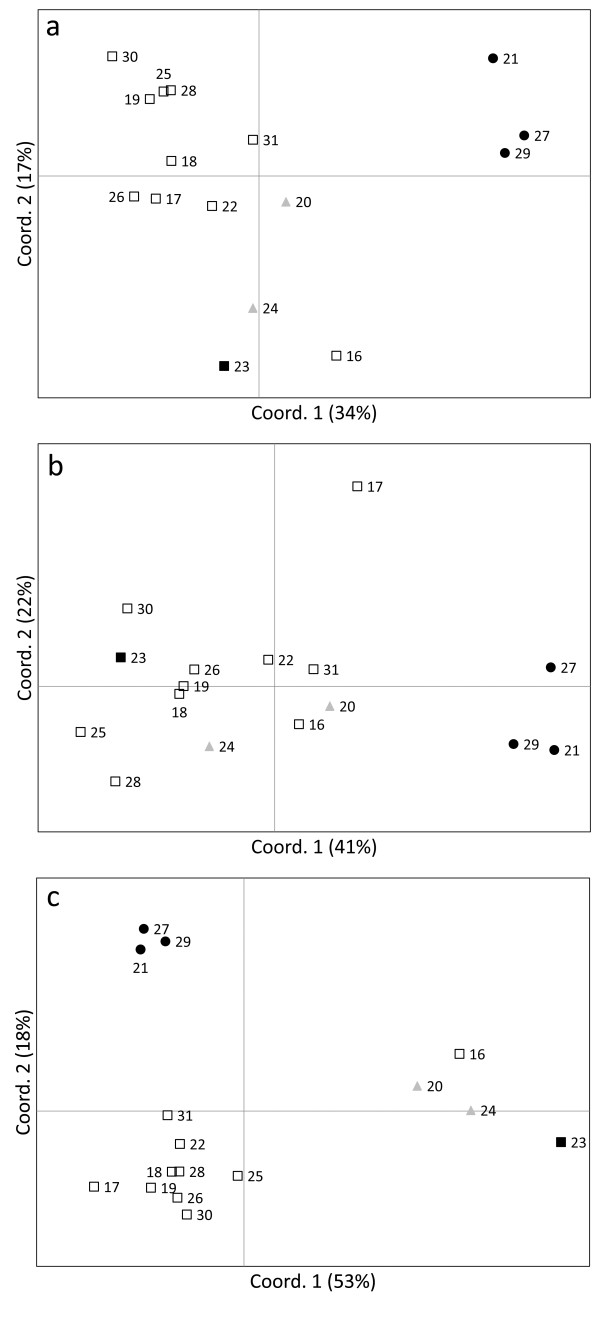
**Principal coordinate analysis (PCO) between sockeye salmon populations from Iliamna Lake**. Based on (a) 38 SNPs ('No Outliers'), (b) 39 SNPs including *One*_*MHC2-190 *('Outlier 1'), or (c) 39 SNPs including *One*_*MHC2-251 *('Outlier 2'). Population numbers can be found in Table 1. Population legends--tributary spawners: empty squares; island-beach spawners: black circles; mainland-beach spawners: grey triangles; Iliamna River (#23, a late-spawning population, Table 1): black square.

Principal coordinate analyses (PCO) from pairwise *F*_ST _within Iliamna Lake showed that spawning habitat explained a great extent of fine-scale clustering patterns (Figure 6a-c). Island beaches (21-Flat Island, 27-Triangle Island, and 29-Woody Island) comprised a genetically homogeneous group (all tests of differentiation: *p *> 0.1; see additional file 1) that was markedly distinct from populations spawning in tributaries and mainland beaches. Tributary spawners comprised a homogeneous cluster despite some exceptions, namely 16-Chinkelyes Creek (Figure 6c), 17-Copper River (Figure 6b) and 23-Iliamna River (Figure 6a, 6c); the latter population spawns later in the season than the majority of Iliamna Lake tributary populations. No significant divergence was evident between mainland-beach sites 20-Finger Beach and 24-Knutson Beach (all tests of differentiation: *p *> 0.1; see additional file 1), and both appeared significantly differentiated from tributaries for 'Outlier 2' with exception of 16-Chinkelyes Creek (Figure 6c), but less for the other two sets (Figure 6a, 6b).

**Figure 7:**
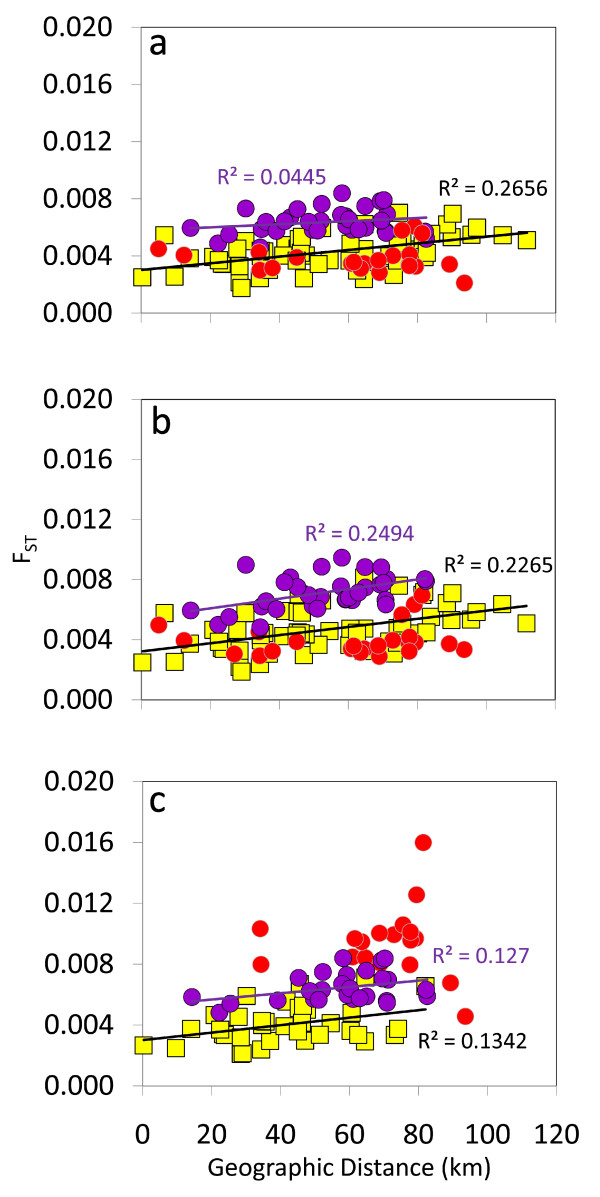
**Scatterplots of genetic (*F*_ST_) vs. geographic distances between sockeye salmon populations from Iliamna Lake**. Scatterplots used (a) 38 SNPs ('No Outliers'), (b) 39 SNPs including *One_MHC2-190 *('Outlier 1'), or (c) 39 SNPs including *One_MHC2-251 *('Outlier 2'). Legend for population comparisons--tributary-tributary: yellow squares; tributary-island beach: purple circles; tributary-mainland beach: red circles. Regression lines and coefficients of determination (R^2^) were only reported for tributary-island beach (purple) and tributary-tributary comparisons (black) as no significant isolation-by-distance patterns were found for tributary-mainland beach comparisons.

We found a significant isolation-by-distance relationship between Iliamna Lake populations even if we ignored habitat differences between them, although determination coefficients were low for all three sets ('No Outliers' *R*^2 ^= 0.06; 'Outlier 1' *R*^2 ^= 0.07; 'Outlier 2' *R*^2 ^= 0.15). These values underwent a two- to five-fold increase once we conducted separate isolation-by-distance analyses for different spawning habitats (Figure 7a-c). Dispersal was thus more likely to occur between tributary populations only than between tributary and island-beach populations, or between tributary and mainland-beach spawners, though only for the 'Outlier 2' set (Figure 7c).

## Discussion

### Hierarchical divergence between Alaskan sockeye salmon populations: the roles of geography and ecology at varying spatial scales

Our premise, that geography and ecology should hierarchically influence population divergence between Kvichak River sockeye salmon populations, was largely supported by various spatial analyses using SNPs. Simple and partial Mantel tests suggested a greater role of geographic distance than differences in spawn timing for the entire drainage. The positive relationship between large-scale genetic and geographic distances was likely driven by discrete genetic differences between lakes as hierarchical AMOVA and the neighbour-joining tree implied: the largest differentiation occurred between populations from distinct lakes followed by differences between populations within lakes. Differences between the two subdrainages in the region (Alagnak and Kvichak) were prominent as well, especially after accounting for the differentiation of Lake Clark.

Large-scale divergence between sockeye salmon populations in the Kvichak River may have initially evolved from historical contingency, followed by contemporary adaptation. Recolonization of lakes and subdrainages was likely sequential as ice sheets retreated after the Late Wisconsin glacial maximum [42]. The best example to illustrate this point is Lake Clark: glacier retreat has only occurred during the last few hundred years in some areas, such as the 13-Upper Tlikakila River [46]. Lake Clark sockeye salmon populations may have therefore become established only very recently. Areas of difficult migratory passage on the Newhalen River below Lake Clark and Sixmile Lake may have also caused bottlenecks during or after the colonization events. Consistent with these findings, Lake Clark populations harboured the lowest estimates of SNP genetic diversity in our study; moreover, an earlier survey using microsatellite DNA indicated widespread presence of bottlenecked populations throughout Lake Clark [46]. Differences in *H*_E _and *A*_R _were also noticeable between the Alagnak and Kvichak subdrainages, which can be attributed to differences in both number and size of populations. For instance, sockeye salmon have historically been much more abundant in the Kvichak than the Alagnak systems [57,58], though recently there has been a surge in abundance among Alagnak populations [59].

Contemporary adaptation to distinct limnetic environments may also help explain the high fidelity of returning adults. Reproductive isolation between nursery lakes is typical of lake-type sockeye salmon, which spend half of their lives rearing in freshwater before migrating to sea [13,44,60-62]. Lake-type sockeye salmon are characterized by a low tendency to disperse, limiting gene flow and enhancing reproductive isolation between populations [14]. Olfactory imprinting during juvenile stages allows lake-type adult salmon to recognize their natal sites [18,19]. In addition to homing, timing of spawning further isolate populations occupying different lakes [49], although its importance may be secondary as suggested by Mantel tests. For instance, Lake Clark populations tend to spawn during early fall, in contrast to populations from Sixmile and Iliamna lakes that spawn from middle to late summer, thus supporting the idea that temporal segregation reduces gene flow [46,47,49,63,64].

The role of ecology (spawn timing and habitat) was more prominent at fine scales or for intralake patterns of divergence, with geographic distance having a significant but secondary role. This conclusion was drawn from analyses conducted within Iliamna Lake, a large and population-rich lake harbouring three different spawning habitats. Mantel tests for one SNP set ('Outlier 2') strongly suggested that variable timing of spawning, particularly among populations of the three spawning-habitat types (see summary in additional file 3), may be the most important barrier to dispersal between these populations. Salmon populations are generally composed of early, late, and even intermediate migrants that breed asynchronously [24,47]. Variable timing of reproduction between spawning-habitat types is thought to be function of historical thermal regimes at the incubation sites of the progeny [23,24]. Temperatures and various other physical attributes vary substantially among tributary, mainland-beach, and island-beach habitats (additional file 3: see also [65-67], which together have promoted locally adapted populations. PCO and isolation-by-distance plots reiterated our expectation that dispersal was more likely to occur between sockeye salmon populations spawning within the same habitat as seen in the Wood River, Alaska [48]. This was particularly striking for the homogeneous group of island beaches as they consistently clustered far from tributaries and mainland beaches. Historical abundances among island-beach populations spanning 45 years of aerial surveys appeared highly correlated [67], which mirrors the genetic homogeneity found in this and other studies [68]. Unique habitat characteristics of island beaches have likely driven this marked divergence (additional file 3). Island-beach populations spawn during an unusually contracted period (about 2 weeks), from early to middle August, in mild wind-circulated water. Temperatures in the range 10 - 13°C allow embryos to hatch prior to freezing in early December; conversely, embryos hatch much later among mainland beaches and tributaries [45]. Island-beach sockeye salmon are also younger and smaller for a given age than tributary sockeye salmon, suggestive of distinct norms of reaction between growth and maturation [45]. Island and mainland beaches further differ in gravel size of incubation sites, which selects for larger eggs among females in island- than mainland-beach spawners [69].

Differences between mainland beaches and tributaries were more obvious for 'Outlier 2' (39 SNPs, including the intronic *One*_*MHC2-251*) than for the other two sets: 'No Outliers' (38 SNPs, excluding *One*_*MHC2-190 *and *One*_*MHC2-251*) suggested mild separation, whereas 'Outlier 1' (39 SNPs, including the exonic *One*_*MHC2-190*) indicated no clear separation. Assuming MHC introns have shorter coalescent times than exons (see following section, *Nonsynonymous and linked SNPs in the MHC class II*), populations from mainland beaches may have diversified only recently from tributaries, and different SNP sets reflect different times since divergence. Consistent with this hypothesis, mainland beach populations appeared undifferentiated from 16-Chinkelyes Creek, a tributary of the Iliamna River, implying that habitat-driven isolation is incomplete. It is possible that populations from 16-Chinkelyes Creek and adjacent tributaries colonized 20-Finger Beach given that only *ca*. 5 km separates it from the mouth of Iliamna River, where 16-Chinkelyes Creek drains. Interestingly, 23-Iliamna River also clustered more closely to mainland beaches than the majority of tributary spawners. We speculate that the four populations from the east of Iliamna Lake (16-Chinkelyes Creek, 20-Finger Beach, 24-Knutson Beach, and 23-Iliamna River) shared a common ancestor. Likely, divergent attributes of their breeding sites as well as discrete patterns of spawn timing have driven them apart over time.

Overall, the fact that geography and ecology have influenced genetic divergence and structure of Kvichak River sockeye salmon in a hierarchical manner has fundamental and applied implications. First, it provides a compelling mechanism for reproductive isolation at varying scales, including isolation-by-distance [50] and isolation-by-time [47]. Also, and because there is substantial phenotypic divergence between spawning ecotypes (additional file 3), it is possible that gene flow is maladaptive between these populations (i.e., immigrants have lower reproductive success than residents). This hypothesis is consistent with findings from another lake system that drains into Bristol Bay, where dispersers between beach and tributary habitats resemble phenotypically their recipient rather than their source populations [21]. Second, it reinforces the importance of maintaining the integrity of all hierarchical levels of intraspecific biodiversity or biocomplexity [9], which have evolved during thousands of years [15], but are currently threatened by anthropogenic changes that have intensified during the last century [12].

### Nonsynonymous and linked SNPs in the MHC class II: evidence for adaptive divergence at the molecular level?

Two SNPs found in one exon (*One_MHC2-190*) and one intron (*One_MHC2-251*) within the MHC class II locus appeared robust to violations of demographic assumptions (e.g., bottlenecks, hierarchical structure) and consistently appeared as outliers during genome scans. MHC class II genes are translated on the surface of antigen-presenting B cells and macrophages and play a key role in the successful mounting of the immune response of vertebrates [70]. Several investigations support the adaptive nature of MHC polymorphisms resulting from pathogen-mediated selection [53,71-74]. Adaptive variation in the MHC is thus likely to affect mate choice, because parents would try to increase pathogen resistance in the offspring, avoid inbreeding, or both [70]. Miller *et al. *[53] concluded that balancing selection is a strong candidate to maintain the allele diversity of the MHC class II locus in Fraser River sockeye salmon (Canada), including the substitution found at *One_MHC2-190 *located in the antigen recognition site. Yet, evidence for diversifying selection or neutrality of alleles could not be ruled out for some populations [53].

We hypothesize that large *F*_ST _estimates for MHC SNPs (*One_MHC2-190 F*_ST _= 0.434; *One_MHC2-251 F*_ST _= 0.387) in comparison with putatively neutral loci are consistent with signatures of diversifying selection; such force is expected to drive adaptive mutations and tightly linked sites to fixation by positive selection, hence increasing differentiation between populations [33]. Kvichak River sockeye salmon populations may have evolved resistance or immunocompetence to specific pathogens that vary in space [53]. Three other studies in salmonids have found signatures of diversifying selection at MHC class I and II gene-linked markers that were also characterized by elevated estimates of population differentiation [75-77].

Even though the argument for diversifying selection seems compelling, it is based on a genome scan involving only 42 nuclear loci, a limitation of most genetic surveys in nonmodel organisms [31,32]. Evaluating alternative hypotheses for the evolution of MHC genes among Kvichak River sockeye salmon populations may be appropriate in the light of some additional findings. In particular, we found that nuclear SNP sets with and without outliers were significantly correlated for the entire drainage. Landry & Bernatchez [78] compared MHC class II and microsatellite alleles for Atlantic salmon (*Salmo salar*) from Central Québec (Canada), and concluded that between-river differentiation was highly correlated between alleles of different marker types, whereas within-river differentiation was not. For Kvichak River sockeye salmon, it is feasible that neutral evolution has played a more prominent role than selection influencing large-scale divergence. Moreover, recent studies argue that the evolution of MHC variation may proceed in neutral fashion: estimates of population divergence from MHC genes appear no different from estimates using neutrally evolving microsatellite DNA markers [79-81].

Could differences between SNP sets found at fine spatial scales be also attributed to selection at the molecular level? Within Iliamna Lake only, differences were evident among 'No Outliers', 'Outlier 1', and 'Outlier 2' sets, including results from PCO and Mantel tests relating spawn timing and genetic distances. These differences were expected if diversifying selection affects those sets containing MHC outliers ('Outlier 1', 'Outlier 2', or both) but not the putatively neutral set ('No Outliers'). The observed dichotomy between sets containing the MHC exon ('Outlier 1') and MHC intron ('Outlier 2') was however unexpected based on the premise that diversifying selection should fix variation in tightly linked sites around the adaptive mutation; here, we have assumed that the MHC exon may have a selective advantage, whereas the MHC intron is subjected to hitchhiking selection [33]. We propose that differences between 'Outlier 1' and 'Outlier 2' may reflect varying coalescent times and thus provide distinct measures of time since population divergence. Introns coalesce more rapidly than exons, because variation in the latter can be maintained by balancing selection, a mechanism that has been demonstrated in MHC exon-intron boundaries where recombination occurs [82,83].

In summary, diversifying selection--as suggested by outlier analyses--acting on two SNPs found in the MHC class II locus complex remains one possible hypothesis with support from other three studies in salmonids. However, neutral evolution of these polymorphisms and impacts of balancing selection represent alternatives that may operate at varying spatial scales. Resolving between these hypotheses is beyond the scope of this investigation; we encourage further studies in sockeye salmon that address mate choice [81] as well as pathogen-host interactions [72-74] to discern between these alternatives.

## Conclusions

Two main conclusions emerge from this study. First, we have demonstrated that geography and ecology have hierarchically influenced genetic divergence between Kvichak River sockeye salmon populations depending on the spatial scale. Contrasts between lakes, subdrainages, and geographic distance dominated large-scale differentiation, whereas differences in the timing of spawning linked to discrete spawning habitat dominated fine-scale (intralake) differentiation. Second, we determined signatures of selection in two SNPs located in the MHC class II that appeared robust to violations of demographic assumptions. We propose that one possible mechanism that has driven the evolution of these SNPs is diversifying selection in response to local pathogens; however, neutral evolution of these polymorphisms at large spatial scales, as well as effects of balancing selection at fine spatial scales, cannot be ruled out at this stage. Both conclusions imply that historical contingency and contemporary adaptation have driven differentiation between Kvichak River sockeye salmon populations, as revealed by a suite of SNPs. Our findings highlight the need for conservation of complex population structure, because it provides resilience in the face of environmental change, both natural and anthropogenic.

## Methods

### Samples and population attributes

Adult sockeye salmon (n = 3,945) were taken from a larger collection of reference populations used for identification of juvenile mixtures in the high seas of the northern Pacific Ocean [41]. Briefly, spawning grounds associated with six major lakes--Battle, Kukaklek, Nonvianuk, Iliamna, Sixmile, and Clark--were surveyed between 1999 and 2006 (Figure 1 and Table 1). Temporal replicates taken in the same location one or more years apart were pooled following earlier assessment guidelines [84], but samples from discrete early- or late-migrant collections were kept separate. Populations were classified according to the type of spawning habitat: mainland beaches, island beaches, or tributaries (Table 1). In both beach types the salmon spawn in the lake itself, but the mainland- and island-beach habitats differ markedly in a number of physical attributes including temperature, gravel size, and flow regime. Consequently, the salmon using these beaches differ in life-history traits such as size at age, age composition, egg size, and spawn timing [45,69]. Habitat characteristics of the three spawning ecotypes and their life-history attributes can be found in additional file 3. We estimated historical dates (day and month) of peak spawning activity (spawn timing, hereafter) as the median of the spawning period reported by Regnart [85] for the Kvichak subdrainage, or the approximate date with the highest live-to-dead fish ratio reported by Clark [57] for the Alagnak subdrainage. Peak spawning dates reported by Ramstad *et al. *[46] were also used as reference for some populations. When spawning periods were unavailable for some populations of the Kvichak subrainage, we calculated the median of the spawning period for the geographic group they belong to. For some late- or early-migrant populations (Table 1) we used the collection date as spawning date.

### Genotyping

Uniplex and array-based genotyping followed Seeb *et al. *[86]. All individuals were genotyped for a panel of 45 SNPs spanning 42 nuclear and three mitochondrial loci [39-41]. Quality control consisted of re-genotyping of 8% of each population to ensure accuracy and reproducibility. Genotyping error was estimated in less than 0.5% [41].

### Detection of outliers (genome scans)

We employed BAYESCAN to identify outliers or those SNPs characterized by higher or lower levels of population divergence than strictly neutral loci, suggestive of diversifying or balancing selection, respectively [51]. BAYESCAN incorporates locus- as well as population-specific regression terms, therefore avoiding unrealistic assumptions of previous methods, such as an island model of migration, symmetrical gene flow, and equal population sizes [31,51]. Prior to simulations, we removed mitochondrial SNPs and monomorphic nuclear SNPs (using a cutoff criterion of >0.98 for the most common allele). Inclusion of monomorphic markers resulted in 40% of our SNPs being outliers, which we considered an unrealistic outcome (authors' unpublished results; see also [31]). After 10 pilot runs of 5000 iterations each, default values of proposed distributions were updated throughout 100,000 Markov Chain Monte Carlo steps after an initial burn-in of 50,000 steps. We assumed chains converged if acceptance rates ranged between 0.25 and 0.45. The top criterion of 'decisive' (log10 Bayes Factor: 2 - 5), which corresponds roughly to a posterior probability range of 0.99 - 1, was indicative that a locus was affected by selection. BAYESCAN simulations were repeated after iteratively removing groups of populations (lakes or subdrainages) to investigate if outliers had a specific geographic origin. This was done in conjunction with allele frequency plots across multiple populations to ascertain geographic trends.

We also used Arlequin 3.5 [52] to detect outlier loci taking into account the hierarchical structure of the Kvichak River, wherein dispersal is likely to be more predominant within subdrainages than between subdrainages. Even though BAYESCAN incorporates population-specific terms, it is unclear whether it takes into account hierarchical structure in its decision-making process. We ran 20,000 simulations assuming 100 demes per group, two hierarchical groups (subdrainages), and a hierarchical island model. These analyses were performed following the exclusion of strongly differentiated populations (Lake Clark) that were flagged in the previous analysis using BAYESCAN.

Outlier loci were classified as exonic or intronic using BLASTX or TBLASTX (National Center for Biotechnology Information) to gauge the scope of selection acting directly on these markers. We followed Smith *et al. *[39] guidelines that considered an alignment significant if an E-value < 10^-5 ^was found.

### Linkage disequilibrium and Hardy-Weinberg equilibrium tests

We tested for deviations from linkage equilibrium between markers in each population using GENEPOP 4.0 [87]. Exact probabilities using a Markov Chain consisting of 100 batches and 5000 iterations per batch were calculated. Correction for multiple tests was done using a sequential Bonferroni correction for multiple *k *tests [88]. For SNPs in significant linkage disequilibrium, we removed the least informative of the pair based on Shannon-Weaver's index (*I*) per locus supplied by GENALEX 6.3 [89]. Linked mitochondrial SNPs were combined into haplotypes following Habicht *et al. *[41] and analyzed separately from nuclear SNPs.

Deviations from Hardy-Weinberg equilibrium among nuclear SNPs were estimated in GENALEX with probabilities for locus-specific χ2 tests. Multilocus HWE goodness-of-fit probabilities were calculated by summing across loci (degrees of freedom = number of loci).

### Genetic diversity and differentiation

Estimation of allele frequencies and heterozygosities (observed and expected) for nuclear SNPs was done in GENALEX. Allelic richness or the number of alleles corrected for sample size was additionally estimated using a rarefaction method implemented in FSTAT 2.9.3 [90,91]. Correlation between allelic richness and expected heterozygosity was judged employing Spearman *R *coefficient in SPSS 17.0. For mitochondrial SNPs, we calculated haplotype frequencies in GENALEX via the AMOVA option for haploid data. Differences in genetic diversity between spatial groups (e.g., subdrainages, lakes) were evaluated using nonparametric Kruskall-Wallis χ^2 ^tests or Mann-Whitney *U*-tests for multiple and pairwise comparisons, respectively, using SPSS 17.0.

For nuclear SNPs, global *F*_ST _plus confidence intervals (95% CI) were obtained from FSTAT, whereas pairwise *F*_ST _values were calculated in GENALEX along with population differentiation tests based on 1000 permutations. Global differentiation tests for *F*_ST _were calculated in GENEPOP using Fisher's method. For mitochondrial SNP haplotypes we quantified global differentiation using Φ_PT _[92] in GENALEX.

### Hierarchical analysis of molecular variance (AMOVA)

We employed the AMOVA option in GENALEX to partition the total genetic variance within and between regions. Hierarchical regions corresponded to either subdrainages or lakes. AMOVA first included populations from the entire drainage; variance components were recalculated after excluding highly differentiated populations to account for an uneven distribution of the total genetic variance. Our objective was to define which grouping explained the highest proportion of the variance. Permutations (1000 times) of elements between and within regions were carried out using Φ-statistics [92] to enable comparisons between nuclear and mitochondrial SNPs.

### Spatial genetic structure

Using PHYLIP [93] we built an unrooted neighbour-joining tree from a matrix of Cavalli-Sforza & Edwards [55] chord distances between populations for the entire drainage. Branch bootstrap support--the percentage each branch appeared in a consensus tree built from 1000 resampled ones--was also estimated; bootstrap support values higher than 50% were reported to highlight those branches that consistently appeared in the bootstrap consensus tree. Branch lengths and tree topologies were visualized in Treeview [94]. Additionally, we used TreeFit [56] to calculate a *R*^2^-value that explains how well the branch lengths of a bifurcating tree captures the variation of the distance matrix. Kalinowski [56] suggests that only trees with *R*^2 ^>0.90 reliably reflect the underlying spatial genetic structure of a distance matrix.

We used a principal coordinate analysis (PCO) in GENALEX to summarize multidimensional genetic data between populations within Iliamna Lake. Pairwise *F*_ST _were preferred at fine scales instead of Cavalli-Sforza & Edwards [51] chord distances. Scores from the first two eigenvectors were plotted, which often accounted for 50% or more of the total variation in the data.

### Testing the relative influence of geographic distance and ecological factors (spawn timing and habitat) on population divergence

Geographic distances (km) corresponded to direct waterway distances calculated in the DeLorme Topo USA^® ^6.0 software (Alagnak subdrainage) or were taken from a spatial analysis of historical abundances among populations of the Kvichak subdrainage [67]. The difference in spawn timing (days) corresponded to the absolute difference between peak spawning dates of two populations. Associations between these variables and genetic distances were tested in the software ZT [95] using simple and partial Mantel tests. Whereas simple Mantel tests can be used to estimate the strength of the correlation between two matrices of distances, partial Mantel tests enable inclusion of a third matrix that is held constant [96]. A partial test may be more informative than a simple Mantel test to gauge the relative importance of the two factors that simultaneously influence genetic structure. Mantel tests were performed for the entire drainage (large scale) and for Iliamna Lake populations only (fine scale), which concentrated the highest number of populations and the greatest diversity of spawning-habitat types. We reported Pearson correlation coefficients (*r*) and their *p*-values after 10000 randomizations. Independence of geographic distances and differences in spawn timing was verified at large scales (entire drainage: *r *= -0.015, *p *= 0.464) and fine scales (Iliamna Lake: *r *= 0.14, *p *= 0.13).

Because spawning-habitat type was a categorical variable, it was not included in Mantel tests. However, we explored whether dispersal gauged through isolation-by-distance patterns [50] was more likely to occur between populations spawning in the same habitat than different habitats (tributary, island beach, and mainland beach) in Iliamna Lake. Because tributary populations outnumbered the other two, we recalculated correlation values between pairwise *F*_ST _and geographic distance for tributary-to-tributary, tributary-to-island-beach, and tributary-to-mainland-beach population comparisons. Linearized (*F*_ST_/1 - *F*_ST_: Rousset *et al. *[97]) or standard pairwise *F*_ST _generated identical results; we thus opted for the latter measure for simplicity.

### Ethics statement

Experimental research reported in this manuscript consisted of genetic analyses of animal tissues collected from wild populations. Collection was done in compliance with protocols from the University of Washington Institutional Animal Care and Use Committee and permits from the Alaska Department of Fish and Game.

## Authors' contributions

DGU conducted the majority of statistical analyses and was the primary responsible for writing the manuscript. JES conceived and designed the study, helped collect many of the samples and interpret the results, and contributed to drafting and editing the manuscript. MJS performed exploratory spatial and genetic analyses and commented on the manuscript. CH participated in the study design, collected and analyzed genetic data, helped interpreting the results, and commented on earlier drafts. TPQ provided ecological expertise on the system, helped collect many of the samples, and contributed to the writing of the manuscript. LWS conceived and designed the study, helped interpret the results, and contributed to drafting and editing the manuscript. All authors read and approved the final manuscript.

## Supplementary Material

Additional file 1 Pairwise *F*_ST _values and tests of differentiation between Kvichak River sockeye salmon populations Click here for file

Additional file 2 Hierarchical analysis of molecular variance (AMOVA) between sockeye salmon populations Click here for file

Additional file 3 Habitat and life-history attributes of three spawning ecotypes of sockeye salmon Click here for file
