# Supplementary material for: Single nucleotide polymorphisms unravel hierarchical divergence and signatures of selection among Alaskan sockeye salmon (Oncorhynchus nerka) populations
Source: BMC Evol Biol. 2011 Feb 18;11:48. doi: 10.1186/1471-2148-11-48 (PMC3049142; doi:10.1186/1471-2148-11-48)
Supplement: Additional file 2 — Hierarchical analysis of molecular variance (AMOVA) between sockeye salmon populations [file 1471-2148-11-48-S2.DOC]

Additional file 2. Hierarchical analysis of molecular variance (AMOVA) between sockeye salmon populations

| Dataset‡ | Hierarchical regions | SNP type | Source* | df | % Variance | *P*-value |
| --- | --- | --- | --- | --- | --- | --- |
|  |  |  |  |  |  |  |
| Entire drainage | Lakes | Nuclear | BR | 5 | 13 | < 0.001 |
|  |  |  | BPWR | 25 | 1 | < 0.001 |
|  |  |  | WP | 3914 | 86 |  |
|  |  | Mitochondrial | BR | 5 | 6 | < 0.001 |
|  |  |  | BPWR | 25 | 1 | < 0.001 |
|  |  |  | WP | 3914 | 93 |  |
|  | Subdrainages | Nuclear | BR | 1 | 8 | < 0.001 |
|  |  |  | BPWR | 29 | 9 | < 0.001 |
|  |  |  | WP | 3914 | 83 |  |
|  |  | Mitochondrial | BR | 1 | 4 | < 0.001 |
|  |  |  | BPWR | 29 | 5 | < 0.001 |
|  |  |  | WP | 3914 | 91 |  |
| Lake Clark excluded | Lakes | Nuclear | BR | 4 | 10 | < 0.001 |
|  |  |  | BPWR | 22 | 1 | < 0.001 |
|  |  |  | WP | 3534 | 89 |  |
|  |  | Mitochondrial | BR | 4 | 6 | < 0.001 |
|  |  |  | BPWR | 22 | 1 | < 0.001 |
|  |  |  | WP | 3534 | 93 |  |
|  | Subdrainages | Nuclear | BR | 1 | 9 | < 0.001 |
|  |  |  | BPWR | 25 | 4 | < 0.001 |
|  |  |  | WP | 3534 | 87 |  |
|  |  | Mitochondrial | BR | 1 | 5 | < 0.001 |
|  |  |  | BPWR | 25 | 2 | < 0.001 |
|  |  |  | WP | 3534 | 93 |  |

‡Because Lake Clark populations were the most divergent, we performed separate analyses with and without these populations. Analyses are based on 38 (putatively neutral) nuclear and 3 mitochondrial SNPs *BR = Between Regions; BPWR = Between Populations Within Regions; WP = Within Populations; df = degrees of freedom.
