# Supplementary material for: Single nucleotide polymorphisms unravel hierarchical divergence and signatures of selection among Alaskan sockeye salmon (Oncorhynchus nerka) populations
Source: BMC Evol Biol. 2011 Feb 18;11:48. doi: 10.1186/1471-2148-11-48 (PMC3049142; doi:10.1186/1471-2148-11-48)
Supplement: Additional file 3 — Habitat and life-history attributes of three spawning ecotypes of sockeye salmon [file 1471-2148-11-48-S3.DOC]

Additional file 3. Habitat and life-history attributes of three spawning ecotypes of sockeye salmon.

|  | Island beach | Mainland beach | Tributary | Reference(s) |
| --- | --- | --- | --- | --- |
|  |  |  |  |  |
| Habitat attributes |  |  |  |  |
| Temperature | 10 - 13°C | 6 - 12°C | Variable | Stewart *et al*. [67] |
| Gravel size (cm) | 7.6 - 30 | 0.3 - 7.5 | 0.3 - 30 | Blair *et al*. [45] |
| Flow regime | wind circulation | upwelling | spring- or glacier-fed | Stewart *et al.* [67] |
| Bear predation | low | low | high | Quinn *et al.* [20] |
|  |  |  |  |  |
| Life-history attributes* |  |  |  |  |
| Mean age at maturity (years; male/female) | 4.44/4.49 | 4.44/4.49 | 4.68/4.74 | Blair *et al.* [45] |
| Average length (mm) | 427 | 455 | 447 - 466 | Quinn *et al.* [69] |
| Mean egg weight (mg) | 115.8 - 116.1 | 91.5 | 90.5 - 110.0 | Quinn *et al.* [69] |
| Fecundity (eggs/female) | 4120 | 3947 | 4210 - 4452 | Blair *et al.* [45] |
| Body depth (mm) | 173.6 – 181.1 | 173.0 | 146.7 – 161.8 | Blair *et al.* [45] |
| Spawn timing‡ | 7 August - 16 August | 20 September - 6 October | 15 August - 17 October | Regnart [85]; this study |

*Only includes data from two island beaches, one mainland beach, and four tributaries from Iliamna Lake.
